# Editorial: Sports immunometabolism, training load, and nutrition: effects on sports performance and psychological behavior of athletes

**DOI:** 10.3389/fpsyg.2023.1253502

**Published:** 2023-10-09

**Authors:** Hadi Nobari, Jason M. Cholewa, Katsuhiko Suzuki

**Affiliations:** ^1^Faculty of Sports Sciences, University of Extremadura, Cáceres, Spain; ^2^Department of Exercise Physiology, College of Health Sciences, University of Lynchburg, Lynchburg, VA, United States; ^3^Faculty of Sport Sciences, Waseda University, Tokorozawa, Japan

**Keywords:** sports immunometabolism, training load, acute response, biochemistry, fatigue, GPS, physical fitness, nutrition

## Introduction

Sports performance is a multifaceted phenomenon influenced by the interactions of various factors, including sports immunometabolism, training load, and nutrition (Lima et al., 2023). These factors contribute to changes in athletes' performance and psychological behaviors during training, matches, and decision-making processes. Physiological factors such as nutrition, heart rate (Silva et al., 2022), hormone secretion (Nobari et al., 2021b), immune system function, and the type of sport, along with psychological factors such as stress, sleep quality, anxiety, and coaches' behavior, play integral roles in shaping athletes' experiences. Understanding the impact of these factors is vital for optimizing athletic performance, preventing injuries and overtraining, and enhancing overall wellbeing. This Research Topic aims to gather scientific evidence that can be translated into practical advice for coaches and athletes, facilitating performance improvement during training and competitions.

## Contributing articles

The articles within this Research Topic address diverse aspects of sports immunometabolism, training load, and nutrition, providing insights into their effects on sports performance and psychological wellbeing. Spanning a range of disciplines, including exercise physiology, nutritional science, psychology, and sports medicine, these studies collectively examine the comprehensive interplay of these factors. By investigating these relationships, the research presented offers valuable knowledge on how athletes can enhance their performance and maintain optimal mental health.

## Immunometabolism and sports performance

Immunometabolism, the study of the metabolic processes underlying immune system function, is a rapidly evolving field with significant implications for sports performance. Several articles in this Research Topic explore the intricate relationship between immune system functioning, energy metabolism, and athletic performance (Suzuki, 2021). These studies highlight the impact of immune markers, such as cytokines and inflammation, on sports performance and recovery. Furthermore, they provide evidence-based interventions for optimizing immunometabolic balance in athletes, enhancing their ability to cope with the physiological demands of training and competition (Nobari et al., 2021a).

## Training load and sports performance

Training load, encompassing the volume, intensity, and duration of physical training, is crucial in determining sports performance outcomes. The articles in this Research Topic investigate various aspects of training load, including its quantification, periodization, and impact on physiological adaptations and performance outcomes. By analyzing the intricate relationship between training load and performance, these studies offer evidence-based strategies for optimizing training programs, preventing overtraining, and maximizing athletic potential. They emphasize the importance of individualized training approaches that consider age, gender, and sport-specific demands.

## Nutrition and sports performance

Proper nutrition serves as a fundamental pillar of sports performance. The articles within this Research Topic examine the impact of nutrition on various aspects of athletic performance and psychological wellbeing. Researchers investigate the roles of macronutrients, micronutrients, hydration, and supplementation in optimizing sports performance. Their findings provide valuable insights into the specific nutritional requirements of athletes, emphasizing the need for individualized approaches tailored to their unique physiological and performance needs. Additionally, these studies explore the effects of nutrition on cognitive function, mood, and mental health, highlighting the bidirectional relationship between nutrition and psychological wellbeing (Bustamante-Sanchez et al., 2022).

## Conclusion

The articles presented in this Research Topic contribute to our understanding of the complex interactions among sports immunometabolism, training load, and nutrition about sports performance and psychological behavior of athletes. By bridging the gaps between multiple disciplines, these studies pave the way for evidence-based interventions and strategies that can enhance athletic performance, prevent injuries, and promote the overall wellbeing of athletes. We are confident that this collection of articles will serve as a valuable resource for researchers, coaches, and practitioners in the field, ultimately benefiting athletes of all levels and disciplines.

## Author contributions

HN: Project administration, Writing—original draft, Writing—review and editing. JC: Writing—original draft, Writing—review and editing. KS: Writing—original draft, Writing—review and editing.
